# L‐type Ca^2+^ channel recovery from inactivation in rabbit atrial myocytes

**DOI:** 10.14814/phy2.15222

**Published:** 2022-03-11

**Authors:** Elizabeth Martinez‐Hernandez, Lothar A. Blatter, Giedrius Kanaporis

**Affiliations:** ^1^ Department of Physiology & Biophysics Rush University Medical Center Chicago Illinois USA

**Keywords:** atria, Ca^2+^ channel, Ca^2+^ cycling, Ca^2+^/calmodulin‐dependent kinase II, recovery from inactivation

## Abstract

Adaptation of the myocardium to varying workloads critically depends on the recovery from inactivation (RFI) of L‐type Ca^2+^ channels (LCCs) which provide the trigger for cardiac contraction. The goal of the present study was a comprehensive investigation of LCC RFI in atrial myocytes. The study was performed on voltage‐clamped rabbit atrial myocytes using a double pulse protocol with variable diastolic intervals in cells held at physiological holding potentials, with intact intracellular Ca^2+^ release, and preserved Na^+^ current and Na^+^/Ca^2+^ exchanger (NCX) activity. We demonstrate that the kinetics of RFI of LCCs are co‐regulated by several factors including resting membrane potential, [Ca^2+^]_i_, Na^+^ influx, and activity of CaMKII. In addition, activation of CaMKII resulted in increased *I*_Ca_ amplitude at higher pacing rates. Pharmacological inhibition of NCX failed to have any significant effect on RFI, indicating that impaired removal of Ca^2+^ by NCX has little effect on LCC recovery. Finally, RFI of intracellular Ca^2+^ release was substantially slower than LCC RFI, suggesting that inactivation kinetics of LCC do not significantly contribute to the beat‐to‐beat refractoriness of SR Ca^2+^ release. The study demonstrates that CaMKII and intracellular Ca^2+^ dynamics play a central role in modulation of LCC activity in atrial myocytes during increased workloads that could have important consequences under pathological conditions such as atrial fibrillations, where Ca^2+^ cycling and CaMKII activity are altered.

## Graphical abstract

Adaptation of the heart to different workloads depends on the recovery from inactivation of the L‐type Ca^2+^ current that serves as the trigger for Ca^2+^ release and cardiac contraction. Unlike in ventricular cells, recovery from inactivation of L‐type Ca^2+^ channels in atrial myocytes has not been investigated in depth. We demonstrate how the kinetics of recovery from inactivation of L‐type Ca^2+^ channels in rabbit atrial myocytes are regulated by resting membrane potential, Ca^2+^/calmodulin‐dependent kinase II, intracellular Ca^2+^ concentration, Na^+^influx, and activity of Na^+^/Ca^2+^ exchanger.

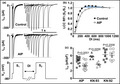

## 1 INTRODUCTION

L‐type Ca^2+^ channels (LCCs) play an essential role for cardiac electrophysiology. L‐type Ca^2+^ current (*I*_Ca_) not only contributes to the generation of the action potential (AP), but also participates in signaling pathways that modulate multiple cellular functions such as mitochondrial energy production, gene transcription, and cell death (Bers, 2008). Most importantly, in cardiac myocytes *I*_Ca_ serves as a trigger for Ca^2+^ release from the sarcoplasmic reticulum (SR) by Ca^2+^‐ induced Ca^2+^ release (CICR) that leads to the activation of myofilaments and contraction of the heart. In cardiac myocytes Ca^2+^ homeostasis is tightly controlled by the regulation of Ca^2+^ influx via *I*_Ca_ and extrusion from the cell by Na^+^/Ca^2+^ exchanger (NCX), as well as Ca^2+^ release from and uptake into the SR. LCC activation by depolarization of membrane voltage (*V*_m_) is followed by their inactivation that is both *V*_m_ and Ca^2+^ dependent. Upon *I*_Ca_ activation and subsequent SR Ca^2+^ release the local [Ca^2+^] in close vicinity of LCCs is elevated which leads to Ca^2+^ binding to calmodulin that is pre‐bound to the channel, resulting in Ca^2+^‐dependent inactivation (CDI) of LCCs (Eckert & Chad, 1984; Pitt et al., 2001; Simms et al., 2014). CDI serves as physiological negative feedback mechanism that limits excess Ca^2+^ entry and stabilizes [Ca^2+^]_i_. Increased pacing frequencies lead to a progressive increase in *I*_Ca_ amplitude, a process known as Ca^2+^‐dependent facilitation (CDF) (Bers & Morotti, 2014; Blaich et al., 2010; Lee et al., 2006; Picht et al., 2007; Yuan & Bers, 1994). Consequently, kinetics of *I*_Ca_ are synergistically governed by both CDF and CDI which allows fine‐tuned adaptation to changing workloads of the heart. Adaptation to changes in work demands also critically depends on the recovery from inactivation (RFI) of *I*_Ca_. RFI refers to the process that readies LCCs for the next heartbeat. Most previous studies have investigated RFI of LCCs in ventricular myocytes providing valuable insights into the mechanisms of RFI regulation. In atrial cells, however, LCC RFI remains poorly investigated. Although regulation of LCCs in atrial and ventricular myocytes share similarities, atrial and ventricular excitation‐contraction coupling (ECC) reveal substantial differences (Blatter et al., 2021), for example, differences in organization and density of t‐tubules (Dobrev, 2017; Huser et al., 1996; Tidball et al., 1991) or different expression of phospholamban resulting in higher sarcoplasmic/endoplasmic reticulum calcium ATPase (SERCA) activity in atria (Boknik et al., 1999). Furthermore, atrial and ventricular cells are each endowed with unique sets of ion channels (Bartos et al., 2015) that leads to distinctive AP morphologies and different *I*_Ca_ kinetics during the AP. Therefore, the goal of the present study was a comprehensive investigation of LCC RFI in rabbit atrial myocytes. Previously it was established that in ventricular myocytes Ca^2+^/calmodulin‐dependent kinase II (CaMKII) plays an essential role in LCC CDF (Bers & Morotti, 2014; Blaich et al., 2010; Lee et al., 2006; Picht et al., 2007; Yuan & Bers, 1994) and RFI (Cheng et al., 2012; Guo & Duff, 2006). While RFI is important for adaptation to higher heart rates under normal conditions, the importance of RFI kinetics becomes even more apparent in pathological conditions that lead to depolarized *V*_m_ and increased cytosolic [Ca^2+^]_i_ that are slowing RFI of LCCs (Altamirano & Bers, 2007). Consequently, impaired RFI contributes to the negative force‐frequency relationship observed under pathophysiological conditions such as heart failure (Antoons et al., 2002). In addition, CaMKII becomes increasingly active in several cardiac pathologies (Anderson et al., 2011; Hegyi et al., 2019; Vincent et al., 2014) and an increased CaMKII activity was associated with enhanced arrhythmogenicity, including atrial fibrillation (Chelu et al., 2009; Yan et al., 2018). CaMKII‐dependent modulation of LCCs increases *I*_Ca_ amplitude (Lee et al., 2006) and accelerates RFI leading to arrhythmias by predisposing to AP prolongation, early afterdepolarizations, delayed afterdepolarizations (Bers & Morotti, 2014), SR Ca^2+^‐leak (Ai et al., 2005), and enhanced inward NCX current (Heijman et al., 2014).

This study demonstrates that the kinetics of RFI of LCCs in atrial myocytes are regulated by several factors including resting *V*_m_, [Ca^2+^]_i_, Na^+^ influx, and activity of CaMKII. In contrast to previous studies (Acsai et al., 2011; Ryu et al., 2012) we have not found any significant contribution of NCX to the recovery of LCCs. Simultaneous recordings of recovery of LCCs and SR Ca^2+^ release indicate that in atrial cells inactivation of LCC and RFI does not significantly contribute to the beat‐to‐beat refractoriness of SR Ca^2+^ release during ECC.

## 2 MATERIALS AND METHODS

### 2.1 Myocyte isolation

Atrial myocytes were isolated from male New Zealand White rabbits (~2.5 kg; Charles River Laboratories (62 rabbits) and Envigo (12 rabbits)). All procedures and protocols were approved by the Institutional Animal Care and Use Committee of Rush University and comply with the Guide for the Care and Use of Laboratory Animals of the National Institutes of Health. Rabbits were anesthetized with an intravenous injection of sodium pentobarbital (100 mg/kg) and heparin (1000 I.U./kg). Hearts were excised, mounted on a Langendorff apparatus and retrogradely perfused via the aorta. After an initial 5 min perfusion with oxygenated Ca^2+^‐free Tyrode solution (in mM: 140 NaCl, 4 KCl, 10 D‐Glucose, 5 Hepes, 1 MgCl_2_, 10 2,3‐butanedione monoxime (BDM), 1000 I.U./l Heparin; pH 7.4 with NaOH), the heart was perfused with minimal essential medium Eagle (MEM) solution containing 20 µM Ca^2+^ and 22.5 µg/ml Liberase TH (Roche Diagnostic Corporation) for ~25 min at 37˚C. The left atrium was dissected from the heart and minced, filtered, and washed in MEM solution containing 50 µM Ca^2+^ and 10 mg/ml bovine serum albumin. Isolated cells were washed and kept in MEM solution with 50 µM Ca^2+^ at room temperature (20–24˚C) and were used within 1–8 h after isolation.

### 2.2 Electrophysiological measurements

*I*_Ca_ was recorded from single atrial myocytes in the whole‐cell ruptured patch clamp configuration using Axopatch 200A and 200B patch clamp amplifiers, the Axon Digidata 1440A, and 1550B interfaces and pCLAMP 10.3 software (Molecular Devices). Current recordings were low‐pass filtered at 5 kHz and digitized at 10 kHz. For *I*_Ca_ measurements patch clamp electrodes were pulled from borosilicate glass capillaries (WPI) with a horizontal puller P‐97 (Sutter Instruments) and filled with internal solution containing (in mM): 130 Cs^+^ glutamate, 10 NaCl, 10 CsCl, 0.33 MgCl_2_, 4 MgATP, and 10 Hepes with pH adjusted to 7.2 with CsOH. Internal solutions were filtered through 0.22‐μm pore filters. Electrode resistance was 1.5–3 MΩ when filled with internal solution. In some experiments 5 mM EGTA or 10 mM BAPTA were added to the internal solution for increased Ca buffering. Nominally applied resting voltage and voltage steps were corrected for a junction potential error of −10 mV. Series resistance was compensated to 70%–80%. The external solution contained (in mM): 135 Na^+^ glutamate, 4 CsCl, 2 CaCl_2_, 1 MgCl_2_, 10 Hepes, 10 D‐glucose, pH 7.4 with NaOH. All experiments were performed at room temperature (20–24°C). For Na^+^‐free experiments all Na^+^ in the external solution was substituted by N‐methyl‐D‐glucamine and in the internal solution NaCl was replaced by CsCl.

Recovery from inactivation was monitored using a two square voltage step (S_1_–S_2_) protocol. Atrial cells were held at −90 mV resting potential and depolarized to 0 mV for 400 ms during S_1_ and S_2_ voltage steps. Both S_1_ and S_2_ steps were preceded by 50 ms prepulses to −60 mV to inactivate Na^+^ current. The time interval between S_1_ and S_2_ steps is termed here as diastolic interval (DI) and was varied from 50 to 1050 ms. Each S_1_–S_2_ pair was preceded by six depolarizing steps to 0 mV for 400 ms at 1 Hz to ensure consistent *I*_Ca_ recordings and SR Ca^2+^ loading. The ratio of *I*_Ca_ amplitudes observed during S_2_ to that evoked by S_1_ (S_2_/S_1_) was used as a measure of the recovery of the channels from inactivation. RFI was quantified by the time constant (τ_RFI_) of S_1_/S_2_ dependence on DI. τ_RFI_ was calculated by fitting recovery curves to a single exponential association equation (GraphPad Prism 5): *Y* = *Y*_0_+ (*Y*_max_ − *Y*_0_)*(1 − *exp(*−*(t *− *t*_0_)/τ_RFI_)) where, *t*_0_ was set to 50 ms, *Y*_max_ was set to ≤1. *Y* represents S_2_/S_1,_ *Y*_0_ is the average S_2_/S_1_ value between *t* = 0 and *t*_0_. The goodness of the fit was assessed by the standard deviation of the residuals $(\text{SDR}=\sqrt{\Sigma \left({\text{residual}}^{2}\right)/\left(n‐k\right)}$; where residual is the vertical distance of a measured data point from the fit line, n is the number of data points, *k* is the number of parameters fit by regression, *n−k* equals to the number of degrees of freedom of the regression). Only τ_RFI_ obtained from fits with SDR < 0.15 were included in the statistical analysis. In addition to τ_RFI_ we also provide time to 50% (*t*_50_) and 80% (*t*_80_) recovery of *I*_Ca_ as a quantitative measure of RFI.

### 2.3 Cytosolic [Ca^2+^] measurements

In a series of experiments [Ca^2+^]_i_ was monitored simultaneously with *I*_Ca_. For [Ca^2+^]_i_ measurements cells were loaded with fluorescent probes Fluo‐4 pentapotassium salt (100 µM, Molecular Probes/Life Technologies) or Cal520 potassium salt (100 µM, AAT Bioquest) via the patch pipette. Fluo‐4 and Cal520 fluorescence were excited at 485 nm with a Xe arc lamp and [Ca^2+^]_i_‐dependent fluorescence signals were collected at 515 nm using a photomultiplier tube. Background‐subtracted fluorescence emission signals (*F*) were normalized to diastolic fluorescence (*F*_0_) recorded under steady‐state conditions at the beginning of an experiment, and changes of [Ca^2+^]_i_ are presented as changes of *F*/*F*_0_. Data recording and digitization were achieved using the Axon Digidata 1440A interface and pCLAMP 10.3 software. Fluorescence signals were low‐pass filtered at 50 Hz.

### 2.4 Chemicals and stock solutions

All chemicals and reagents were from MiliporeSigma unless otherwise stated. Stock solutions used: aqueous solutions of 1 mM Autocamtide‐2‐Related Inhibitory Peptide (AIP, Calbiochem) and 10 mM tetrodoxin (TTX, Bio‐Techne), while 200 mM lidocaine, 2 mM YM‐244769, and 50 mM ORM‐10103 stock solutions were prepared in DMSO.

### 2.5 Data analysis and presentation

Results are presented as individual observations and as mean ± SEM. n represents the number of individual cells and *N* is the number of rabbits. Statistical difference between data sets was evaluated using Welch *t*‐test or paired *t*‐test and differences were considered significant at *p *< 0.05. τ_RFI_ data of control and BAPTA experiments at different *V*_h_ were compared using Brown–Forsythe one‐way ANOVA test followed by the post hoc group comparison by Benjamini–Krieger–Yekutieli test.

## 3 RESULTS

### 3.1 Effect of CaMKII inhibition on RFI of LCCs

**FIGURE 1:**
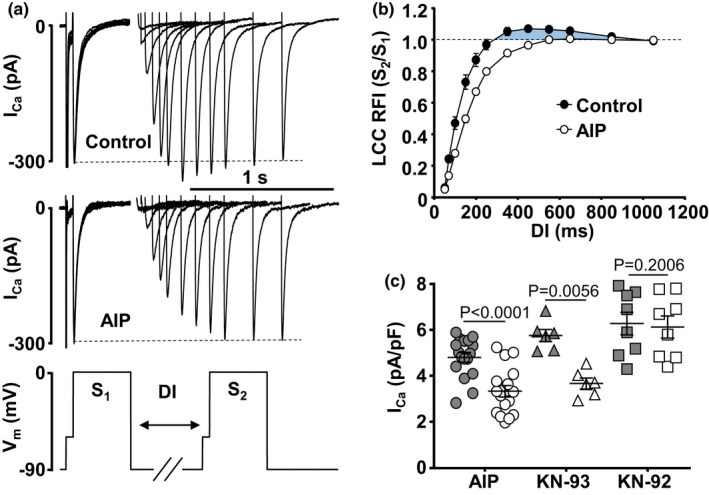
Inhibition of CaMKII slows LCC RFI. (a) Family of *I*_Ca_ traces elicited in atrial myocytes with a double pulse S_1_–S_2_ voltage protocol (bottom) in control and in the presence of 1 µM AIP for the diastolic interval (DI) range of 50 to 1050 ms. CaMKII inhibition slowed LCC RFI kinetics and abolished the LCC RFI overshoot at DI = 250–650 ms. *I*_Ca_ more negative than the dashed line indicates *I*_Ca_ overshoot. (b) LCC RFI curves. Average S_2_/S_1_ ratios of *I*_Ca_ amplitudes recorded during S_2_ and S_1_ depolarization steps in control (*n*/*N* = 43/24) and in cells dialyzed with AIP (*n*/*N* = 17/6) as a function of DI. CaMKII inhibition abolished LCC RFI overshoot (shaded area). (c) Peak *I*_Ca_ amplitudes recorded in control (grey symbols) and in the presence of CaMKII inhibitors (open symbols) AIP (1 µM, *n*/*N* = 17/6) and KN‐93 (1 µM, *n*/*N* = 6/3) in atrial cells paced at 1 Hz. KN‐92 (1 µM, *n*/*N* = 8/4), an inactive analog of KN‐93, had no significant effect on LCC amplitude. Data presented as mean ± SEM. Statistical analysis was performed with Welch *t*‐test for AIP data and paired *t*‐test for KN‐93 and KN‐92

CaMKII was proposed to play an important role in the LCC recovery from inactivation in ventricular myocytes (Cheng et al., 2012; Guo & Duff, 2006), however such data for atrial myocytes are lacking. To investigate RFI of LCCs in atrial myocytes (Figure 1), using the whole‐cell voltage clamp technique, we applied a double depolarization pulse (S_1_–S_2_) protocol (Figure 1a). A control pulse (S_1_) to 0 mV was applied from a holding potential (*V*_h_) of −90 mV for 400 ms and was preceded by a brief (50 ms) prepulse to −60 mV to keep voltage‐gated Na^+^ channels in the inactivated state during S_1_ and thus prevent “contamination” of *I*_Ca_ by *I*_Na_. S_1_ was followed by the same test voltage step (S_2_) applied at variable intervals after S_1_. The interval between S_1_ and S_2_ (referred to here as diastolic interval, DI) was varied from 50 to 1050 ms. Peak *I*_Ca_ measured during the S_2_ pulse was normalized to peak *I*_Ca_ elicited by the S_1_ pulse (S_2_/S_1_ ratio). The rate of *I*_Ca_ RFI was quantified by: (1) the S_2_/S_1_ ratio as a function of DI and (2) by the time constant τ_RFI_ and time to 50% and 80% of *I*_Ca_ recovery (*t*_50_, *t*_80_; summarized in Tables 1 and 2) obtained from single exponential fits of the S_2_/S_1_‐DI curves of individual cells. Figure 1b shows S_2_/S_1_ ratios as a function of DI. In control conditions, *I*_Ca_ recovery revealed an overshoot (S_2_/S_1_ > 1) for DIs ranging between 250 and 650 ms (shaded area in Figure 1b). The largest average overshoot in LCC recovery was observed at a DI of 450 ms where *I_Ca_* increased to 107 ± 2% (*n* = 43, *p* = 0.003) during S_2_ compared to *I*_Ca_ elicited by the S_1_ pulse.

**FIGURE 2:**
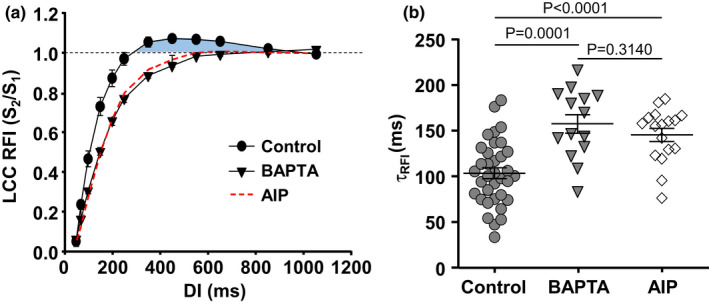
Effect of [Ca^2+^]_i_ on LCC RFI. (a) Average LCC RFI curves recorded in control (*n*/*N* = 43/24) and in cells dialyzed with 10 mM BAPTA (*n*/*N* = 14/6). Dashed curve shows LCC recovery in the presence of 1 µM AIP (from Figure 1b). Data presented as mean ± SEM. (b) Time constants of LCC RFI (τ_RFI_) derived from mono‐exponential fits of individual LCC RFI curves in control and in cells dialyzed with BAPTA and AIP. Data presented as mean ± SEM and analyzed with Welch *t*‐test

To test the effect of CaMKII‐dependent modulation of RFI of LCCs, CaMKII was blocked with 1 µM AIP (delivered to the cell via patch pipette, and a minimum of 3 min of cell dialysis with internal solution was allowed before *I*_Ca_ recordings). AIP reduced peak *I*_Ca_, slowed RFI kinetics and completely abolished the overshoot of *I*_Ca_ recovery (Figure 1b), suggesting that the overshoot is a manifestation of CaMKII elicited Ca‐dependent facilitation of *I*_Ca_. Addition of 1 µM AIP to the internal solution resulted in a reduction of peak *I*_Ca_ (measured 3 min after whole patch configuration was established) from 4.81 ± 0.21 pA/pF in control (*n *= 17) to 3.33 ± 0.24 pA/pF (*n* = 17, *p *< 0.0001) (Figure 1c). This observation is similar to previous reports in ventricular myocytes (Huang et al., 2016) and with our results obtained using an alternative CaMKII blocker (KN‐93, 1 µM) where *I*_Ca_ amplitude decreased from 5.76 ± 0.26 pA/pF in control to 3.67 ± 0.23 pA/pF (*n *= 6, *p *= 0.0056) in the presence of KN93, while the inactive analogue of KN‐93, KN‐92 (1 µM) had no statistically significant effect on LCC amplitude (control: 6.28 ± 0.49 pA/pF; KN‐92: 6.13 ± 0.48 pA/pF; *n *= 8, *p *= 0.2006, Figure 1c). In the presence of AIP recovery of LCCs was significantly slower (Figure 2a) and τ_RFI_ increased from 104 ± 6 ms (*n* = 37) in control to 147 ± 7 ms (*n *= 17; *p *< 0.0001) (Figure 2b). *t*_50_ and *t*_80_ in the presence of AIP are shown in Table 1. Figures 1 and 2 show that CaMKII inhibition has profound effects on *I*_Ca_ magnitude and RFI.

**TABLE 1:** τ_RFI_, *t*_50_ and *t*_80_ (mean ± SEM) of LCC RFI in control and various experimental conditions

Condition	τ_RFI_ (ms)	*t*_50_ (ms)	*t*_80_ (ms)	*n*
Control	104 ± 6	63 ± 4	150 ± 9	37
AIP	147 ± 7	95 ± 5	224 ± 10	17
EGTA	123 ± 17	80 ± 11	191 ± 25	9
BAPTA	157 ± 10	97 ± 5	235 ± 13	14
Lidocaine	191 ± 19	122 ± 11	280 ± 23	8
Na‐free	308 ± 27	187 ± 13	436 ± 29	14
TTX	131 ± 17	84 ± 10	204 ± 24	7
ORM‐10103	91 ± 20	56 ± 13	132 ± 31	7
YM‐244769	103 ± 15	62 ± 10	154 ± 26	5

### 3.2 Effect of intracellular Ca^2+^ buffering on RFI of LCCs

Because of the Ca^2+^ dependence of CaMKII action, we tested the effect of intracellular Ca^2+^ buffering on LCC recovery from inactivation (Figure 2). Addition of 10 mM BAPTA to the internal pipette solution slowed RFI of LCCs and abolished the overshoot, similar to the CaMKII inhibitor AIP, confirming that the overshoot is [Ca^2+^]_i_ dependent and is consistent with CDF of *I*_Ca_. τ_RFI_ of LCC increased to 157 ± 10 ms (*n *= 14, *p *< 0.0001 vs. control) in the presence of BAPTA (Figure 2b). The slowed *t*_50_ and *t*_80_ values in the presence of BAPTA are shown in Table 1. Mean *I*_Ca_ amplitude in the presence of BAPTA was 6.28 ± 0.59 pA/pF and, due to lack of CDI, tended to be slightly larger than in control cells (4.98 ± 0.26 pA/pF; *n* = 43; *p *= 0.0620 Welch *t*‐test). Qualitatively similar results were found with the weaker and slower Ca buffer EGTA (5 mM; *I*_Ca_ peak 5.57 ± 0.43 pA/pF (*n* = 9) data not shown) that increased τ_RFI_ of LCC to 123 ± 17 ms (*n *= 9, *p *= 0.1997 vs. control) and also abolished the *I*_Ca_ RFI overshoot. Results show that cytosolic Ca^2+^ buffers slowed RFI of LCCs in a similar fashion as CaMKII inhibition with AIP. These results suggest that CaMKII and beat‐to‐beat oscillations in [Ca^2+^]_i_ in atrial myocytes play an important role in LCC adaptation to changes in heart rate by permitting enhanced Ca^2+^ influx at faster heart rates through shortening of LCC refractoriness.

### 3.3 Holding potential and RFI of LCCs

**FIGURE 3:**
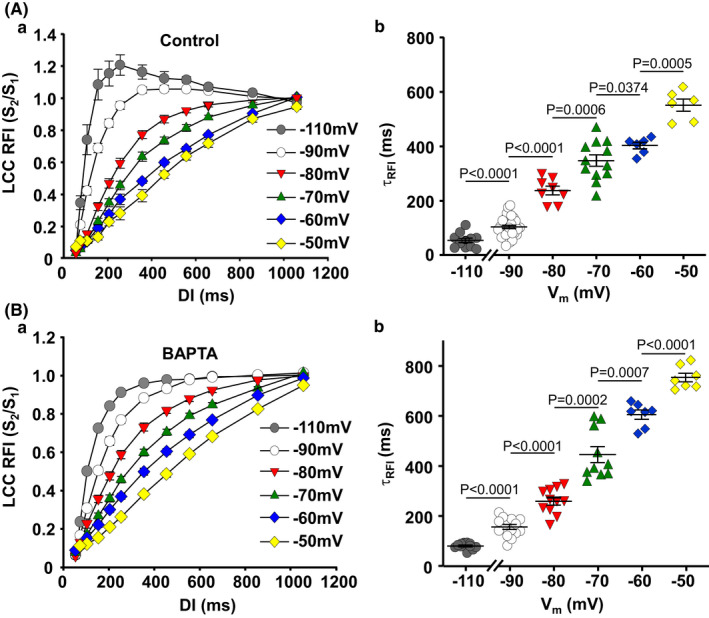
*V*_h_ dependence of LCC RFI. (A) Atrial myocytes LCC RFI curves (a) and individual time constants (τ_RFI_) (b) at holding potentials of −110 (*n*/*N* = 12/9), −90 (*n*/*N* = 37/24), −80 (*n*/*N* = 8/6), −70 (*n*/*N* = 12/8), −60 (*n*/*N* = 6/4), and −50 mV (*n*/*N* = 6/4). (B) *V*_h_‐dependence of LCC RFI curves (a) and τ_RFI_ (b) recorded from individual atrial myocytes with increased intracellular Ca^2+^ buffering by internal perfusion with 10 mM BAPTA at holding potentials of −110 (*n*/*N* = 12/5), −90 (*n*/*N* = 14/6), −80 (*n*/*N* = 11/5), −70 (*n*/*N* = 10/4), −60 (*n*/*N* = 7/4), and −50 mV (*n*/*N* = 7/4). Data presented as mean ± SEM analyzed with Brown–Forsythe one‐way ANOVA test followed by the post hoc group comparison with Benjamini–Krieger–Yekutieli test

In ventricular myocytes the kinetics of RFI of LCCs have been reported to depend on *V*_h_ (Li et al., 1997; Namiki et al., 2007), however *V*_h_ dependence in mammalian atria has not been investigated. Figure 3A shows a strong *V*_h_ dependence of RFI kinetics of *I*_Ca_ in atrial myocytes. With increased degree of *V*_h_ depolarization in the range from −110 to −50 mV LCC recovery kinetics became increasingly slower and τ of RFI increased approximately six fold at −50 mV (Figure 3Ab). RFI dependence on *V*_h_ was also observed in cells with enhanced intracellular Ca^2+^ buffering by BAPTA (10 mM) (Figure 3B) and EGTA (data not shown). The data indicate that *V*_h_ dependence of LCC recovery is not determined by [Ca^2+^]_i_ while overall RFI of *I*_Ca_ was slower in the presence of BAPTA over the range of *V*_h_ tested. τ_RFI_, *t*_50_ and *t*_80_ values are presented in Table 2.

**TABLE 2:** τ_RFI,_ *t*_50_ and *t*_80_ (mean ± SEM; ms) of LCC RFI for *V*_h_ between −110 and −50 mV in control and in presence of BAPTA (10 mM)

	τ_RFI_ (ms) (*n*)		*t*_50_ (ms)		*t*_80_ (ms)	
*V*_h_ (mV)	Control	BAPTA	Control	BAPTA	Control	BAPTA
−110	54 ± 8 (12)	82 ± 3 (12)	32 ± 5	52 ± 2	78 ± 13	128 ± 5
−90	104 ± 6 (37)	157 ± 10 (14)	63 ± 4	97 ± 5	150 ± 9	235 ± 13
−80	238 ± 16 (8)	260 ± 15 (11)	158 ± 11	161 ± 11	364 ± 23	387 ± 23
−70	347 ± 21 (12)	446 ± 31 (10)	232 ± 14	227 ± 9	533 ± 32	540 ± 19
−60	402 ± 12 (6)	605 ± 18 (7)	244 ± 9	287 ± 12	591 ± 13	687 ± 23
−50	551 ± 22 (6)	753 ± 17 (7)	361 ± 18	375 ± 13	835 ± 38	874 ± 27

### 3.4 Effect of *I*_Na_ inhibition on RFI of LCCs

**FIGURE 4:**
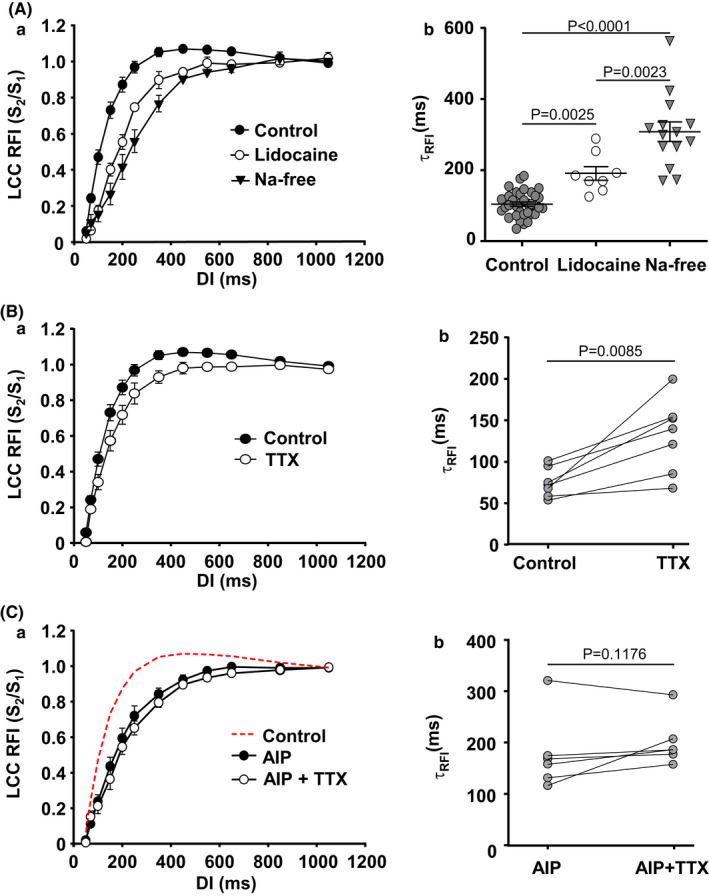
Effect of *I*_Na_ inhibition on LCC RFI. A: (a) LCC RFI curves (mean ± SEM) recorded in control (*n*/*N* = 43/24) and in atrial cells treated with 500 µM lidocaine (*n*/*N* = 8/4) or in Na^+^‐free conditions (*n*/*N* = 14/9). (b) τ_RFI_ (mean ± SEM) from individual LCC RFI curves for control, lidocaine (500 µM), and Na^+^‐free conditions. Means compared with Welch *t*‐test. B: LCC RFI curves (a) and τ_RFI_ (b) recorded before and after exposure to 1 µM TTX (*n*/*N* = 7/3). Means compared with paired *t*‐test. C: (a) Application of TTX (1 µM, *n*/*N* = 6/2) has no significant effect on LCC RFI in the presence of 1 µM AIP. (b) τ_RFI_ recorded from individual atrial myocytes dialyzed with AIP before and after application of TTX. Means compared with paired *t*‐test

Under our control conditions *I*_Na_ was not suppressed allowing physiological Na^+^ influx and normal activity of NCX. In order to compare our results with previous studies in which RFI of LCC was investigated under conditions of full or partial *I*_Na_ inhibition, we performed a series of experiments by blocking *I*_Na_ with lidocaine or TTX (partial *I*_Na_ inhibition with 1 µM of TTX) or eliminating *I*_Na_ by Na^+^ substitution in external and internal solutions with N‐Methyl‐D‐glucamine and CsCl, respectively. Figure 4A shows that elimination of Na^+^ from the internal and external solutions (which also prevents Ca^2+^ extrusion via NCX) resulted in impaired LCC RFI with significantly slower kinetics. Mean *I*_Ca_ density in Na‐free conditions was 4.86 ± 0.26 pA/pF (*n* = 14) and was essentially identical with *I*_Ca_ in control (4.98 ± 0.26 pA/pF, *n* = 43; *p* = 0.7529, Welch *t*‐ test). Additionally, slower RFI kinetics were also observed in cells where *I*_Na_ was suppressed by lidocaine (500 µM, *n* = 8) (Figure 4A). Mean τ_RFI_ was 308 ± 27 ms in Na^+^‐free conditions (*n* = 14) and 191 ± 19 ms (*n* = 8, Table 1) in lidocaine, that is, recovery of *I*_Ca_ was significantly slower than in control (104 ± 6 ms; *n* = 37). Similarly, kinetics of RFI were also slowed by the application of 1 µM TTX. TTX at 1 µM is expected to block late *I*_Na_ and to have just a partial inhibitory effect on peak *I*_Na_ (30%–50%) (Carmeliet, 1987; Kaufmann et al., 2013; Satin et al., 1992). Figure 4B shows paired data of LCC recovery in control and after the application of 1 µM TTX. Incomplete *I*_Na_ block likely accounts for a lesser increase of τ_RFI_ (control: 68 ± 8 ms; TTX: 131 ± 17 ms; *n* = 7) compared with effects of Na^+^‐free conditions or lidocaine application. The mean *I*_Ca_ densities in control and in presence of TTX (paired measurements) were 6.56 ± 0.66 and 5.76 ± 0.74 pA/pF, respectively. Lidocaine at concentrations used here could potentially have a minor blocking effect on LCCs (Josephson, 1988), and thus affect recovery of *I*_Ca_. However, mean *I*_Ca_ density in lidocaine was 5.36 ± 0.89 pA/pF, that is, comparable to control *I*_Ca_ (4.98 ± 0.26 pA/pF, *n* = 43; *p* = 0.7190, Welch *t*‐test). Also, the effects of lidocaine were in‐line with observations in Na‐free conditions and TTX results, thus a potential lidocaine effect on LCCs is unlikely to affect our conclusions.

We also tested for a possible interplay between CaMKII and *I*_Na_ inhibition with TTX. In cardiomyocytes dialyzed with the CaMKII blocker AIP, 1 µM TTX failed to further slow RFI (Figure 4C). In this series of experiments AIP alone increased τ of LCC recovery to 184 ± 34 ms (*n* = 6, *I*_Ca_ amplitude 5.37 ± 0.63 pA/pF). Subsequent exposure to TTX (1 µM TTX, *I*_Ca_ amplitude 4.48 ± 0.70 pA/pF) resulted in τ_RFI_ of 210 ± 26 ms which was not significantly different from the effect of AIP alone (*p* = 0.1176). In summary, our results show that both pharmacological blocker of Na^+^ channels and substitution of Na^+^ resulted in slower LCC recovery.

Pharmacological Na channel inhibition and Na^+^ substitution can also affect NCX function. Therefore, in the next set of experiments we explored a potential contribution of NCX to RFI directly.

### 3.5 NCX inhibition and RFI of LCC

**FIGURE 5:**
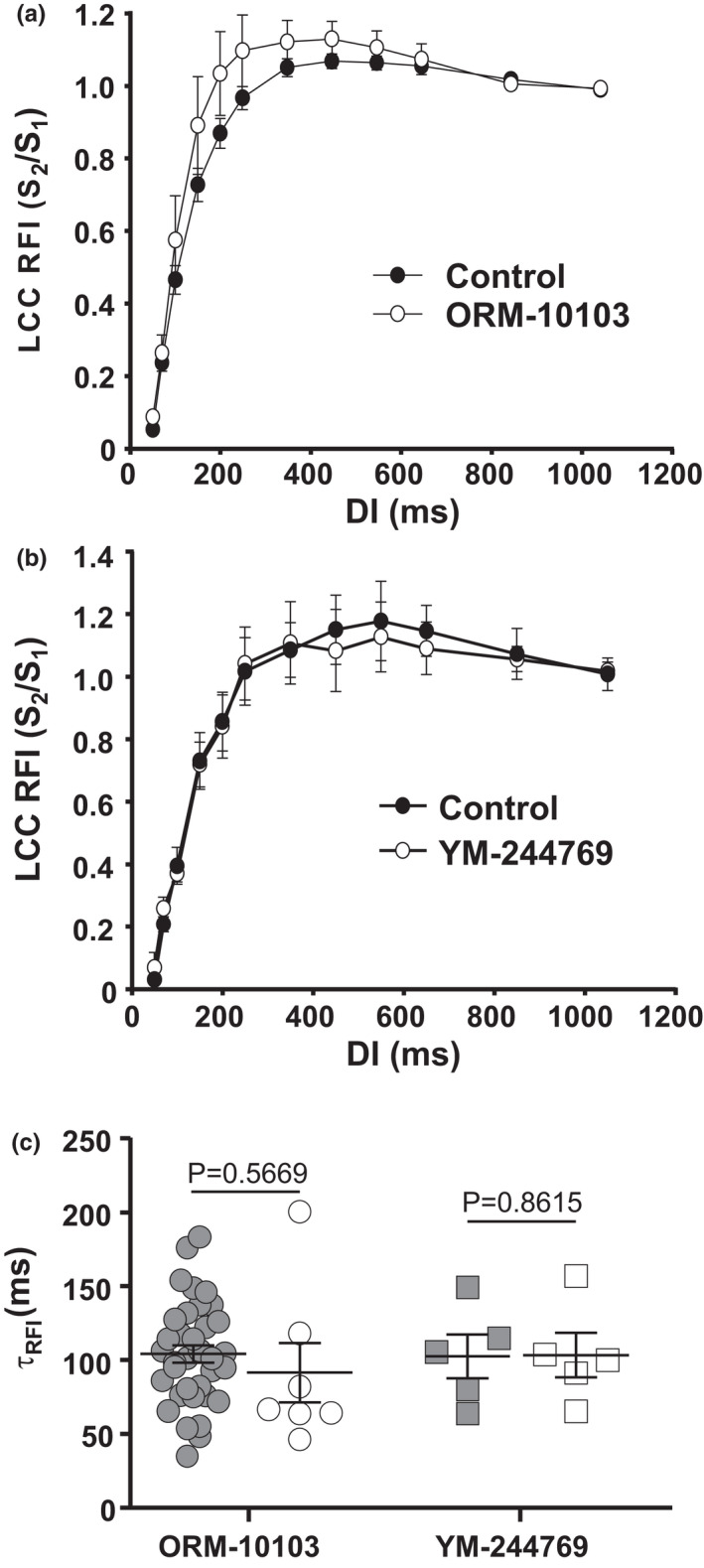
Effect of NCX inhibition on LCC RFI. LCC RFI curves recorded in control and after >30 min incubation with (a) ORM‐10103 (10 µM, *n*/*N* = 7/3), and (b) reverse mode NCX blocker YM‐244769 (400 nM, *n*/*N* = 5/4). Data presented as mean ± SEM. (c) Summary of τ_RFI_ in control and in the presence of NCX inhibitors ORM‐10103 and YM‐244769. Data presented as mean ± SEM and compared with Welch *t*‐test

Previously it was suggested that LCC recovery in ventricular myocytes might be modulated by the activity of NCX (Namiki et al., 2007; Ryu et al., 2012). Our observation found that substitution of Na^+^ substantially slowed recovery of LCCs indirectly suggests the possibility of an involvement of NCX. Therefore, to further investigate the effect of NCX activity on RFI of LCCs we used pharmacological NCX blockers. Preincubation of atrial myocytes with the NCX blocker ORM‐10103 (10 µM, >30 min, *n* = 7; Figure 5a), which inhibits both inward and outward NCX currents (Jost et al., 2013), had no statistically significant effect on τ_RFI_ of LCCs (Figure 5c, Table 1). However, LCC RFI curves in the presence of ORM had a tendency to exhibit a bigger overshoot (to 1.13 ± 0.04 vs. 1.07 ± 0.02 at DI of 450 ms, *p* = 0.1069) which is likely a consequence of increased [Ca^2+^]_i_ and enhanced CDF of *I*_Ca_ (Figure 5a). Furthermore, Figure 5b shows RFI of LCCs in control and after a 3–5 min exposure to the NCX blocker YM‐244769 (400 nM; *n* = 5) which preferentially blocks reverse mode of NCX (Yamashita et al., 2016). YM‐244769 also had no significant effect on RFI kinetics. The time constants are similar for both NCX inhibitors and not significantly different from control (Figure 5c). Therefore, we conclude that in atrial myocytes Ca^2+^ removal by NCX might have a lesser role in modulating RFI than previously suggested (Acsai et al., 2011; Ryu et al., 2012).

### 3.6 Recovery from inactivation of LCC and CaT

**FIGURE 6:**
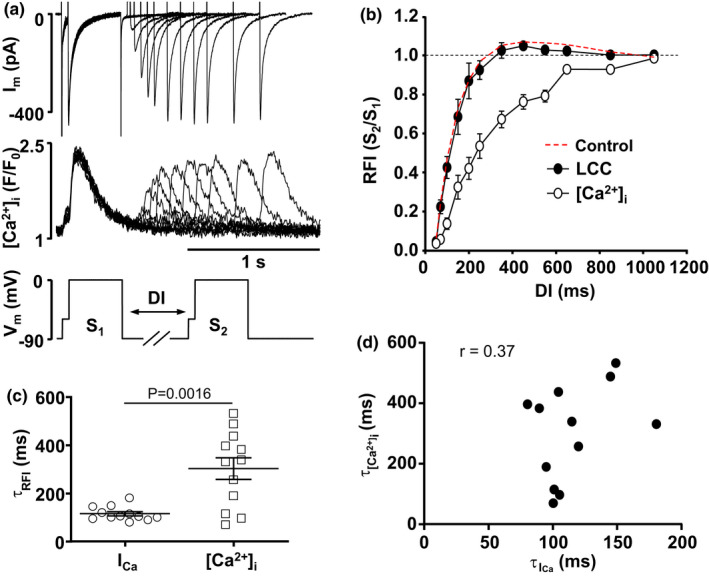
RFI of SR Ca^2+^ release. (a) Simultaneously recorded *I*_Ca_ and [Ca^2+^]_i_ traces during S_1_–S_2_ voltage protocol (bottom). Prepulse depolarization to −60 mV. DI range: 50–1050 ms. (b) LCC and SR Ca^2+^ release (CaT amplitude) RFI curves obtained simultaneously from the same set of atrial myocytes (*n*/*N* = 12/9). Dashed line shows average LCC RFI curve from all control myocytes (*n*/*N* = 43/24). (c) Distribution and mean ± SEM of τ_RFI_ from individual LCC and SR Ca^2+^ release RFI curves. Means compared with Welch *t*‐test. (d) Correlation between LCC and SR Ca^2+^ release τ_RFI_. *r* = Pearson correlation coefficient

In a subset of cells we simultaneously measured *I*_Ca_ and [Ca^2+^]_i_ to compare RFI kinetics of *I*_Ca_ and recovery from refractoriness of SR Ca^2+^ release (Figure 6). RFI of CaTs (Figure 6a) was quantified by normalizing the CaT amplitude elicited by S_2_ to the amplitude triggered by S_1_ (Figure 6b). Kinetics of RFI of *I*_Ca_ were significantly faster than the recovery of intracellular Ca^2+^ release (Figure 6c). In this set of experiments the average τ of RFI of LCCs was 115 ± 8 ms (*n* = 12) while the average τ of CaT recovery was 302 ± 45 ms (*n* = 12). Furthermore, at the level of individual cells only a weak correlation was found between the time constants of CaT and LCC RFI (Figure 6d). These results suggest that, similarly to ventricular myocytes, recovery of intracellular Ca^2+^ release in atria is little affected by inactivation of LCCs as these channels start to recover from inactivation at considerably shorter DIs. Consequently, recovery of CaTs is mostly governed by the rate of SR refilling with Ca^2+^ and/or the refractoriness of the SR Ca^2+^ release mechanism, and not RFI of the primary trigger mechanism of CICR (*I*_Ca_).

## 4 DISCUSSION

### 4.1 LCC RFI in atrial myocytes: effects of [Ca^2+^]_i_ and CaMKII

Kinetics of RFI of LCCs is an important factor determining beat‐to‐beat availability of I_Ca_, especially at increased pacing frequencies. LCC RFI was extensively studied in ventricular myocytes, however, much less is known about the specific properties of LCC recovery in atrial cells. While ECC in atria and ventricular myocytes share similarities, there are also substantial differences. First, atrial and ventricular cells differ in t‐tubule network organization and density. T‐tubules, invaginations of plasma membrane that penetrate deep into ventricular cells, permit rapid AP propagation into the cell interior and assure cell wide synchronous Ca^2+^ release. In atria, however, the t‐tubule system is not well developed or even entirely lacking, and the location of LCCs is generally restricted to the cell periphery (Frisk et al., 2014; Huser et al., 1996; Schulson et al., 2011). This leads to spatio‐temporal inhomogeneities of CICR, where membrane depolarization induces Ca^2+^ release first in sub‐sarcolemmal regions from where CICR subsequently propagates to the center of the cell. In addition, atrial cells have lower expression levels of the endogenous SERCA inhibitor phospholamban (Luss et al., 1999). Finally, ventricular and atrial myocytes differ in their endowment with and activity of surface membrane ion channels such as small conductance Ca^2+^‐activated K^+^ channels (Tuteja et al., 2005) and Ca^2+^‐activated Cl^−^ channels (Kanaporis & Blatter, 2016a, 2016b; Szigeti et al., 1998), while acetylcholine‐activated and ultrarapid rectifier K^+^ channels are expressed exclusively in the atria (Dobrzynski et al., 2001). Unique ion channel expression patterns lead to distinctive AP morphologies that profoundly affect activation and kinetics of LCC (Kanaporis & Blatter, 2017; Sah et al., 2003).

A straightforward comparison of ventricular and atrial RFI properties is also hampered by the different experimental conditions under which investigations were performed. The majority of the studies in ventricular myocytes were conducted in cells with suppressed SR Ca^2+^ release by adding Ca^2+^ chelators to the pipette solution or applying SR Ca^2+^ release blockers. In this study we aimed to investigate LCC RFI under more physiological conditions, that is, we applied a physiological holding membrane potential of −90 mV, Na^+^ currents were not blocked to enable Ca^2+^ extrusion through NCX and the majority of experiments were performed with no or low intracellular Ca^2+^ buffering to permit normal SR Ca^2+^ release. Under similar conditions (with exception for TTX used to inactivate *I*_Na_) Namiki et al. (Namiki et al., 2007) reported comparable RFI recovery kinetics (τ_RFI_ = ~100 ms) in rabbit ventricular myocytes. In addition, consistent with our observations (Figure 1) this study showed an overshoot of the LCC RFI at a DI of ~500 ms. Similarly, an overshoot in RFI recovery was reported in dog and guinea pig ventricular myocytes, where it was enhanced with elevated extracellular [Ca^2+^], and eliminated by increased intracellular Ca^2+^ buffering or block of SR Ca^2+^ release (Tseng, 1988). Here, we demonstrate that an overshoot in LCC RFI is prevented not only by increased intracellular Ca^2+^ buffering (Figure 2), but also by CaMKII inhibition (Figure 1), suggesting that the overshoot is a manifestation of CDF of LCCs. Interestingly, when rabbit ventricular myocytes were stimulated with AP voltage commands (at 37°C) no overshoot in LCC RFI was detected (Altamirano & Bers, 2007). The RFI overshoot is expected to have a significant physiological role in adaptation to increased heart rates as it would allow to sustain adequate Ca^2+^ influx during shortened APs and counteract effects of CDI of LCCs due to elevated diastolic [Ca^2+^]_i_. *I*_Ca_ overshoot was not observed in the previous studies performed under experimental conditions where intracellular Ca^2+^ release was not preserved. Nonetheless, these studies demonstrated that genetic knockdown (Cheng et al., 2012; Xu et al., 2010) or pharmacologic inhibition of CaMKII (Guo & Duff, 2006; Huang et al., 2016; Li et al., 1997; Picht et al., 2007; Vinogradova et al., 2000) delayed LCC recovery and provided strong evidence that CaMKII plays an important role in modulating RFI in ventricular myocytes.

### 4.2 Role of NCX

To investigate *I*_Ca_ properties with the patch clamp technique, ideally all other currents that potentially could contaminate *I*_Ca,_ should be eliminated. Therefore, the vast majority of studies studying RFI of LCCs use at least one of the following approaches to eliminate *I*_Na_: (a) use of depolarized holding potentials of −60 to −40 mV that completely inactivates *I*_Na_, however RFI of LCCs is *V*_m_ dependent and recovery at depolarized *V*_h_ is considerably slower (Jeziorski et al., 2000; Namiki et al., 2007) (Figure 3); (b) Na^+^ substitution in external and internal solutions which also blocks Ca^2+^ extrusion by NCX; or (c) use of pharmacological *I*_Na_ blockers. In this study we applied 50 ms prepulses to −60 mV during which *I*_Na_ is activated and subsequently Na^+^ channels remain refractory during the *I_Ca_* recordings. This approach has some benefits as it allows to investigate *I*_Ca_ properties using physiological holding potentials, and with no pharmacological *I*_Na_ blocker present potential unwanted unspecific drug effects are precluded. [Ca^2+^] in the dyadic cleft is governed by several processes including Ca^2+^ influx through LCCs, CICR from SR, intracellular Ca^2+^ buffering, Ca^2+^ diffusion, Ca^2+^ reuptake into the SR, and Ca^2+^ removal by NCX (Acsai et al., 2011). The role of NCX in LCC RFI has remained unresolved. It was suggested that diminished extrusion of Ca^2+^ by NCX at depolarized potentials contributes to slower RFI (Namiki et al., 2007). In addition, Ryu et al. have proposed that differences in LCC recovery observed in rabbit pulmonary vein cardiac myocytes with and without Na^+^ in the external solutions result from NCX blockage and suggested that NCX plays a critical role in LCC RFI regulation (Ryu et al., 2012). Results of our study, however, do not support the same conclusion. Direct inhibition of NCX with pharmacological blockers ORM‐10103 or YM‐244769 did not slow down LCC RFI (Figure 5). Therefore, we conclude that in atrial myocytes Ca^2+^ extrusion by NCX plays a lesser role in modulating RFI than previously suggested in ventricular and pulmonary vein myocytes (Acsai et al., 2011; Ryu et al., 2012).

### 4.3 LCC recovery versus SR Ca^2+^ release recovery

LCC RFI in ventricular myocytes was demonstrated to be faster than recovery of intracellular Ca^2+^ release (Sun et al., 2018; Wei et al., 2021). Here, we report a similar observation in atrial cells (Figure 6). In addition, no correlation was found between RFI time constants for CaTs and LCCs, suggesting that recovery of LCCs, being considerably faster, is not playing a major role for the slow recovery of SR Ca^2+^ release. This observation supports the notion that refractoriness of SR Ca^2+^ release, which has been linked to development of pro‐arrhythmic cardiac alternans (Kornyeyev et al., 2012; Lugo et al., 2014; Shkryl et al., 2012; Wang et al., 2014) as well as spatial and temporal heterogeneity throughout the myocardium stems from the time limiting process of Ca^2+^ uptake by SERCA and/or the refractoriness the SR Ca^2+^ release machinery (Shkryl et al., 2012). Nonetheless, as discussed above, kinetics of LCC RFI play a critical role for efficient Ca^2+^ release during ECC, and its fine‐tuned regulation is especially critical at higher heart rates.

## 5 CONCLUSIONS

The goal of the present study was to characterize the mechanism of RFI of LCCs in atrial myocytes. The main novel findings are as follows: (1) with *I*_Na_ and intracellular Ca^2+^ release active, CaMKII accelerated recovery of LCC and elicited potentiation of LCCs during DIs ranging from 250 to 650 ms; (2) LCC RFI was slowed by intracellular Ca^2+^ buffering and depended on resting membrane potential; (3) suppression of *I*_Na_ by intra‐ and extracellular Na^+^ substitution or using *I*_Na_ blockers led to a substantial slowing of RFI of LCCs; (4) at variance with previous reports that removal of Ca^2+^ by NCX modulates LCC RFI, NCX blockers used in our study failed to have any significant effect on RFI, indicating that impaired removal of Ca^2+^ by NCX has little effect on LCC recovery. Finally, (5) recovery from inactivation of intracellular Ca^2+^ release was substantially slower than that of LCCs, suggesting that in atrial cells inactivation kinetics of LCC do not significantly contribute to the refractoriness of SR Ca^2+^ release.

## CONFLICT OF INTEREST

The authors declare that there is no conflict of interest.

## AUTHOR CONTRIBUTIONS

E.M‐H, L.A.B, and G.K contributed to conception and design of the experiments, analysis and interpretation of the results. E.M‐H and G.K collected data. E.M‐H, L.A.B, and G.K contributed to writing of the manuscript and approved the final version of the manuscript.
